# Chemotherapy induced histopathological changes in retinoblastoma, assessment of high risk predictive factors & its correlation with comorbid conditions

**DOI:** 10.12669/pjms.38.ICON-2022.5786

**Published:** 2022-01

**Authors:** Nausheen Yaqoob, Salima Mansoor, Nida Zia, Kanwal Aftab, Bushra Kaleem, Saba Jamal

**Affiliations:** 1 Nausheen Yaqoob, FCPS, Department of Histopathology, Indus Hospital & Health Network, Korangi, Karachi, Pakistan; 2 Salima Mansoor, MBBS, Department of Histopathology, Indus Hospital & Health Network, Korangi, Karachi, Pakistan; 3 Nida Zia, FCPS. Department of Pediatric Oncology, Indus Hospital & Health Network, Korangi, Karachi, Pakistan; 4 Kanwal Aftab, FCPS, Department of Histopathology, Indus Hospital & Health Network, Korangi, Karachi, Pakistan; 5 Bushra Kaleem, MPhil (Haematology), Indus Health Research Center, Indus Hospital & Health Network, Korangi, Karachi, Pakistan; 6 Saba Jamal, Diplomate American Board of Haematology, Diplomate American Board of Anatomic and Clinical Pathology, Department of Histopathology, Indus Hospital & Health Network, Korangi, Karachi, Pakistan

**Keywords:** Retinoblastoma, high risk histopathological features, chemotherapy, survival

## Abstract

**Background & Objectives:**

Retinoblastoma is a malignant intraocular tumor and its treatment requires a multidisciplinary approach. Chemotherapy is an important modality in treatment of retinoblastoma. The purpose of this study was to assess the histopathological changes in retinoblastomas treated with chemotherapy along with correlation of comorbid conditions with high risk histopathological factors (HRF).

**Methods:**

All post-chemotherapy enucleated eye specimens received in the pathology department between 2017 to 2021 were included in the study. Slides were retrieved and reviewed for chemotherapeutic effects, tumor regression, and for assessment of HRF. Patient demographic data, information regarding chemotherapy and co-morbid conditions were retrieved through the hospital database. Chi-square was used to analyze the relation between comorbid conditions and HRF.

**Results:**

Chemotherapeutic effects were seen in all eyes with varying degrees of responses. Necrosis, calcification, and gliosis were the most common findings. The majority of eyes showed tumor occupying less than 50% of the eye whereas complete regression was noted in one eye only. Retinal detachment, glaucoma, and buphthalmos were the most common comorbid conditions at the time of diagnosis. Patients with glaucoma were more likely to have ciliary body invasion. Kaplan Meier analysis showed that patients with more than two HRF had decreased survival rates in comparison to those with one or no HRF.

**Conclusion:**

Histopathological evaluation of chemotherapy-treated eyes shows varying degrees of response to chemotherapy. Post enucleation histopathological evaluation of the globe plays an important role in assessing disease activity and guiding further treatment to prevent metastasis.

## INTRODUCTION

Retinoblastoma is a malignant intraocular tumor of the retina occurring due to the exuberant proliferation of retinoblasts. It accounts for 3% of all pediatric tumors.^1^ The average age at diagnosis is 18 months. Leukocoria (white reflex) is the most common symptom at presentation. Other findings include vision loss, cataract, vitreous and sub-retinal seedlings, strabismus, neovascularization of iris, glaucoma, vitreous hemorrhage, and pseudohypopyon. Proptosis can occur in advanced cases.

In the 1960s, initial classification of retinoblastoma was introduced by Reese and Ellsworth to evaluate the success of radiotherapy. This classification assessed tumor size, location, and multifocality. Following the introduction of chemotherapy, this classification was no longer helpful in predicting response to chemotherapy. The Reese Ellsworth classification was replaced by the International Classification of Retinoblastoma (ICRB) in 2003. This classification predicts globe outcome after chemo reduction.^2^,^3^ According to the ICRB classification, tumors are grouped from A to E, based on their size and the extent of tumor seeds in the subretinal space and vitreous cavity.

Several options are available for the treatment of retinoblastoma including enucleation, systemic chemotherapy, external beam radiation, and local treatment options like cryotherapy, transpupillary thermotherapy, and laser. The primary goal of treatment in unilateral retinoblastoma is patient survival and prevention of extraocular dissemination of tumor. Preservation of visual function and protection of the eye are secondary goals in unilateral retinoblastoma. Treatment of retinoblastoma is individualized based on several factors including the ICRB group, laterality of tumor, and vision potential.

Historically, external beam radiation therapy and enucleation were common modalities of treatment. Chemotherapy has recently become an important modality to avoid enucleation. Chemotherapy helps to reduce the size of the tumor (chemoreduction) thereby facilitating local treatment measures like cryotherapy, laser, etc. to control the tumor. It is also used as an adjuvant therapy to decrease the risk of metastasis following enucleation in high-risk patients. There are various routes of administering chemotherapy which include intravenous, intravitreal, intra-arterial, and subconjunctival. Vincristine, Etoposide, and Carboplatin are the standard agents for systemic chemotherapy because of good intraocular penetration.

With recent treatment modalities, it is possible to salvage eyes with group A, B, C, and some group D intraocular tumors, however group E and extraocular tumors require enucleation.^4^,^5^ The treatment for extraocular disease includes chemotherapy followed by enucleation. Group A to C eyes can be salvaged in more than 90 percent of cases. Group D eyes have a lower chance of successful salvage and eyes can be salvaged in 47 percent of cases.^6^

Following enucleation, histopathological assessment of the globe is very important as the histopathology report helps ocular oncologists in deciding further course of treatment. There are few studies on the histopathological findings in retinoblastoma primarily treated with chemotherapy. Histopathological features that are considered as high risk include massive choroidal invasion, invasion of iris, ciliary body, sclera, optic nerve invasion posterior to lamina cribrosa and to cut end of the optic nerve. Some researchers also consider anterior chamber invasion as a high-risk factor but it is still debatable.^7^ This study will focus on histopathological features in chemotherapy-treated eyes along with the correlation of ocular co-morbidities with high-risk histopathological factors.

## METHODS

All patients with retinoblastoma who received chemotherapy only were selected for the study. Enucleated eye specimens received in the pathology department between September 1^st^, 2017 to February 28^th^, 2021 were retrieved for review.

Patient demographic data and information regarding chemotherapy regimen, number of chemotherapy cycles given, ICRB classification, and indication for enucleation were retrieved through Health Management Information System. The slides were reviewed for tumor regression and histopathological changes in the tumor. Status of retinoblastoma was noted as tumor occupying greater than 50% of the eye, less than 50% of the eye, less than 5% or completely regressed. Invasion of eye structures was noted including invasion of the anterior chamber of eye, iris, ciliary body, minimal (tumor <3mm in diameter) and massive (tumor >3mm in diameter) choroidal invasion, scleral/extrascleral invasion, and optic nerve invasion anterior to lamina cribrosa, at lamina cribrosa, posterior to lamina cribrosa and to the cut end of the optic nerve. Only anterior chamber invasion, massive choroidal invasion, scleral invasion, optic nerve invasion posterior to lamina cribrosa and to cut end of the optic nerve were considered as high-risk histopathological features. Other features like necrosis, calcifications, gliosis and retinal detachment were also reviewed. Necrosis and calcification were graded as extensive (>50%), moderate (25-50%), and minimal (<25%). Chi-square test was used to analyze the association between comorbid conditions and invasive disease. KM analysis of study participants based on the number of high-risk factors was also done.

### Ethical Approval:

An approval from the Review Board of Indus Hospital and Health Network was obtained for this study (IHHN_IRB_2021_03_006).

## RESULTS

A total of 77 eyes were received in the histopathology department between September 1st, 2017 and February 28th, 2021. Out of these, 54 underwent primary enucleation whereas 21 received chemotherapy followed by enucleation. The median age of patients was 36 months (IQR: 24-48 months). The median time from diagnosis to enucleation was 2 months (IQR: 1 to 42 months). Demographic data, clinical and histopathological findings at the time of diagnosis are given in Table-I.

**Table I:** Characteristics of study participants.

Variables	N (%)
**DEMOGRAPHIC CHARACTERISTICS**	
Age:	
Median (IQR)	36 months (24 – 48 months)
Gender:	
(M/F)	13 (61.9)/8 (38.1)
Time from diagnosis to enucleation, months:	
Median (IQR)	2 (1 – 42)
No. of patients received chemotherapy:	
CEV	17 (80.9)
EORB	1 (4.8)
CEV + EORB	3 (14.3)
Overall survival:	
N (%)	16 (76.2)
Days of survival:	
Median (IQR)	434 (266.5 - 658)
**CLINICAL CHARACTERISTICS**	
Clinical classification	
• Group D	6 (28.6)
• Group E	10 (47.6)
• Extraocular	5 (23.8)
Tumor stage:	
• pT0	1/21 (4.8)
• pT1	7/21 (33.3)
• pT2	4/21 (19.0))
• pT3	3/21 (14.2)
• pT4	5/21 (23.8)
Cannot be determined	1/21 (4.8)
Tumor grade:	
• G1	3/21 (14.3)
• G2	7/21 (33.3)
• G3	6/21 (28.6)
• G4	1/21 (4.8)
Cannot be determined	4/21 (19.0)
Vitreous seeds	
• Present	7/21 (33.3)
• Absent	14/21 (66.6)
Cataracts	
• Present	0/21 (0)
• Absent	21/21 (100)
Glaucoma	
• Present	4/21(19.0)
• Absent	17/21(80.9)
Retinal detachment	
• Present	7/21 (33.3)
• Absent	14/21 (66.6)
Pthisis bulbi	
• Present	0/21 (0)
• Absent	21/21 (100)
Iris neovascularization	
• Present	2/21 (9.5)
• Absent	19/21 (90.4)
Bupthalmos	
• Present	3/21 (14.2)
• Absent	18/21 (85.7)
Proptosis	
• Present	1/21 (4.7)
• Absent	20/21 (95.2)
Pseudohypopyon	
• Present	1/21 (4.7)
• Absent	20/21 (95.2)
**HISTOLOGICAL FINDINGS**	
Tumor status:	
• >50%	7/21 (33.3)
• <50%	3/21 (14.2)
• <5%	10/21 (47.6)
• Complete regression	1/21 (4.7)
Calcification	
• Extensive	7/21 (33.3)
• Moderate	2/21 (9.5)
• Minimal	12/21 (57.1)
Necrosis	
• Extensive	11/21 (52.3)
• Moderate	2/21 (9.5)
• Minimal	8/21 (38.0)
Gliosis	
• Present	8/21 (38.0)
• Absent	13/21 (61.9)
Rosettes	
• Present	7/21 (33.33)
• Absent	14/21 (66.66)
Optic nerve invasion	
• Pre-laminar	2/21 (9.5)
• Laminar	3/21 (14.3)
• Post-laminar	2/21 (9.5)
• To cut end of optic nerve	4/21 (19.0)
• Tumor free	9/21 (42.9)
• Cannot be determined	1/21 (4.8)
Choroid invasion	
• Minimal	2/21 (9.5)
• Massive	4/21 (19.0)
• Tumor free	14/21 (66.7)
• Cannot be determined	1/21 (4.8)
Scleral invasion	
• Present	3/21 (14.3)
• Absent	17/21 (80.9)
• Cannot be determined	1/21 (4.8)
Iris invasion	
• Present	6/21 (28.6)
• Absent	15/21 (71.4)
Ciliary body invasion	
• Present	5/21 (23.8)
• Absent	15/21 (71.4)
• Cannot be determined	1/21 (4.7)
Anterior chamber invasion	
• Present	6/21 (28.5)
• Absent	15/21 (71.4)

CEV-Carboplatin/Etoposide/Vincristine; EORB-Extra-ocular retinoblastoma chemotherapy.

Of those who received chemotherapy, the majority of eyes were group E (10 eyes, 47.6 %) according to ICRB classification, six eyes (28.6 %) were group D and five (23.8%) had extraocular disease. Indication of enucleation was group E and extraocular disease in 15 eyes and treatment failure in six group D eyes. Nine patients had bilateral retinoblastoma and 12 patients had unilateral retinoblastoma. As per our institutional policy, bilateral retinoblastoma patients, with one advanced eye (Group E) were treated with two cycles of systemic chemotherapy for tumor reduction before enucleation. Group D patients were treated with chemotherapy initially followed by enucleation in case of progression or treatment failure. Most patients received intravenous chemotherapy with a three-drug regimen of Carboplatin (600mg/m^2^ on Day 1), Etoposide (150mg/m^2^ on Days 1 & 2), and Vincristine (1.5mg/m^2^ on Day 1). Four patients received chemotherapy for extraocular Retinoblastoma which included Carboplatin (200mg/m^2^ Days 1, 2 & 3), Etoposide (150mg/m^2^ on Days 1, 2 & 3), Doxorubicin (50mg/m^2^ on Day 1), and Cyclophosphamide (450mg/m^2^ on Days 1, 2 & 3). The majority of patients received two cycles of chemotherapy prior to enucleation. Three patients also received intravitreal Melphalan.

Histopathologic evidence of chemotherapeutic effects was seen in all eyes showing varying degrees of necrosis and calcification. Histopathologic findings in eight (38%) eyes showed tumor regression to less than five percent. Complete regression of the tumor was seen in one eye. Rosettes were present in 33.3% eyes. In terms of HRFs involving the optic nerve, the tumor was still present up to the cut end in 19% patients while 42.9% were found to tumor free. The tumor was completely absent in 66.7% of patients with previously present choroidal invasion while massive invasion of the choroid was still present in 19% patients. Iris and ciliary body invasion was still observed in the post-chemotherapy period in 28.6% and 23.8% patients respectively.

**Fig.1:**
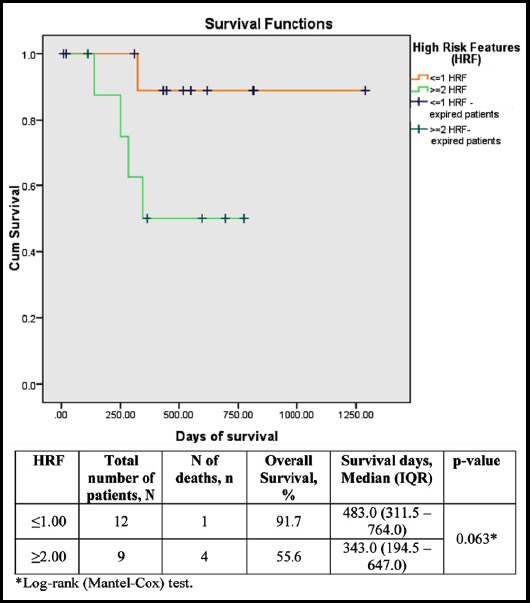
Kaplan-Meier analysis of the study participants on the basis of number of HRFs

A correlation was also done between co-morbid conditions and the presence of high-risk histopathological features shown in Table-II. The presence of glaucoma was found to be significantly associated with the presence of ciliary body invasion (p-value: 0.037). Fifteen patients were alive and five died as of April 2021. One patient was lost to follow up. Kaplan Meier analysis of study participants based on the number of high risk factors is displayed in Fig.1. Patients with two or more high risk factors had low survival rates compared to patients with one or no high risk factors, however, this was not statically significant.

**Table II:** Association between tumor invasion and various comorbidities.

	Optic nerve invasion			Massive choroidal invasion		Minimal choroidal invasion			Iris invasion			Ciliary body invasion			Scleral invasion			

	Yes	No	p-value	Yes	No	p-value	Yes	No	p-value	Yes	No	p-value	Yes	No	p-value	Yes	No	p-value
Time to enucleation																		
≤3 Months	8/14 (57.1)	6/14 (42.9)	0.574	3/14 (21.4)	11/14 (78.6)	0.657	2/14 (14.3)	12/14 (85.7)	1.000	5/14 (35.7)	9/14 (64.3)	0.613	4/13 (30.8)	9/13 (69.2)	1.000	2/14 (14.3)	12/14 (85.7)	1.000
> 3 Months	3/6 (50.0)	3/6 (50.0)		1/6 (16.7)	5/6 (83.3)		-	6/6 (100)		1/6 (16.7)	5/6 (83.3)		1/6 (16.7)	5/6 (83.3)		1/6 (16.7)	5/6 (83.3)	
Necrosis:																		
Extensive	4/8 (50.0)	4/8 (50.0)	1.000	2/8 (25.0)	6/8 (75.0)	1.000	1/8 (12.5)	7/8 (87.5)	1.000	3/8 (37.5)	5/8 (62.5)	0.631	3/8 (37.5)	5/8 (62.5)	0.347	1/8 (12.5)	7/8 (87.5)	1.000
Minimal/Moderate	7/13 (53.8)	6/13 (46.2)		2/12 (16.7)	10/12 (83.3)		1/12 (8.3)	11/12 (91.7)		3/13 (23.1)	10/13 (76.9)		2/12 (16.7)	10/12 (83.3)		3/13 (23.1)	10/13 (76.9)	
Glaucoma:																		
Yes	2/4 (50.0)	2/4 (50.0)	1.000	1/4 (25.0)	3/4 (75.0)	1.000	-	3/3 (100)	1.000	3/4 (75.0)	1/4 (25.0)	0.061	3/4 (75.0)	1/4 (25.0)	0.037*	1/4 (25.0)	3/4 (75.0)	0.509
No	9/16 (56.3)	7/16 (43.8)		3/16 (18.8)	13/16 (81.3)		2/17 (11.8)	15/17 (88.2)		3/16 (18.8)	13/16 (81.3)		2/15 (13.3)	13/15 (86.7)		2/16 (12.5)	14/16 (87.5)	
Pseudohypopyon:																		
Yes	1/1 (100)	-	1.000	-	1/1 (100)	1.000	-	1/1 (100)	1.000	1/1 (100)	-	0.286	1/1 (100)	-	0.250	-	1/1 (100)	1.000
No	10/20 (50.0)	10/20 (50.0)		4/19 (21.1)	15/19 (78.9)		2/19 (10.5)	17/19 (89.5)		5/20 (25.0)	15/20 (75.0)		4/19 (21.1)	15/19 (78.9)		4/20 (20.0)	16/20 (80.0)	
Vitreous seeds:																		
Yes	4/7 (57.1)	3/7 (42.9)	1.000	-	6/6 (100)	0.267	2/6 (33.3)	4/6 (66.7)	0.079	2/7 (28.6)	4/14 (71.4)	1.000	1/6 (16.7)	5/6 (83.3)	1.000	1/7 (14.3)	6/7 (85.7)	1.000
No	7/14 (50.0)	7/14 (50.0)		4/14 (28.6)	10/14 (71.4)		14/14 (100)	4/14 (28.6)		10/14 (71.4)	4/14 (28.6)		10/14 (71.4)	3/14 (21.4)		11/14 (78.6)		
Retinal detachment:																		
Yes	4/7 (57.1)	3/7 (42.9)	1.000	-	7/7 (100)	0.249	2/7 (28.6)	5/7 (71.4)	0.111	2/7 (28.6)	5/7 (71.4)	1.000	2/7 (28.6)	5/7 (71.4)	1.000	-	7/7 (100)	0.255
No	7/14 (50.0)	7/14 (50.0)		4/13 (30.8)	9/13 (69.2)		13/13 (100)	4/14 (28.6)		10/14 (71.4)	3/13 (23.1)		10/13 (76.9)	4/14 (28.6)		10/14 (71.4)		
Iris neovascularization:																		
Yes	2/2 (100)	-	0.476	-	2/2 (100)	1.000	-	2/2 (100)	1.000	2/2 (100)	-	0.071	2/2 (100)	-	0.053	2/2 (100)	-	1.000
No	9/19 (47.7)	10/19 (52.6)		4/18 (22.2)	14/18 (77.8)		2/18 (11.1)	16/18 (88.9)		4/19 (21.1)	15/19 (78.9)		3/18 (16.7)	15/18 (83.3)		4/19 (21.1)	15/19 (78.9)	
Buphthalmos																		
Yes	1/2 (50.0)	1/2 (50.0)	1.000	2/3 (66.7)	1/3 (33.3)	0.088	-	4/4 (100)	1.000	2/3 (66.7)	1/3 (33.3)	0.202	2/3 (66.7)	3/16 (18.8)	0.155	1/3 (33.3)	2/3 (66.7)	0.404
No	10/18 (55.6)	8/18 (44.4)		2/17 (11.8)	15/17 (88.2)		2/16 (12.5)	14/16 (87.5)		4/17(23.5)	13/17 (76.5)		1/3 (33.3)	13/16 (81.3)		2/17 (11.8)	15/17 (88.2)	

## DISCUSSION

The treatment of Retinoblastoma is complex and is in constant evolution. It requires a multidisciplinary approach with the involvement of pediatric oncologists, ophthalmologists, and radiation oncologists. The treatment varies worldwide but the primary goal remains the same i.e. to preserve life, prevent metastatic disease, and salvage the globe and vision whenever possible. Systemic chemotherapy is either given for chemo reduction (reduce tumor size via chemotherapy) or used as adjuvant therapy. Its effect is usually seen after two cycles of chemotherapy with an average reduction of 50% in tumor thickness.^8^ Despite the development of more conservative approaches, approximately 20-30% of patients with bilateral retinoblastoma require enucleation.^9^ The evaluation of the tumor status in the post-chemotherapy period is of immense importance as it helps in assessing the extent of viable tumor as well as the presence of HRFs. It further helps in predicting extraocular dissemination of tumor, the risk stratification of the patient and thus the need for neoadjuvant therapy to reduce the risk of metastasis. Current study evaluates the response to chemotherapy, and the presence of HRFs for future treatment strategy in retinoblastoma patients. Further the presence of co-morbidities was associated with the HRFs as certain features such as retinal detachment have resulted in optic nerve invasion of the tumor.

Histopathological evaluation of the eyes in this study showed that the tumor exhibited variable response to chemotherapy with complete regression of the tumor in one eye while no significant tumor regression (tumor occupying >50% of eye) was seen in 33.3% eyes as shown in Table-I. Enucleation is decided on the baseline grouping of the eye. Groups D and E are non-salvageable and thus have to be enucleated with no option of globe salvage. However, post-enucleation histopathology is important to further designate the management plan to decrease the metastatic potential, so as to improve the survival. According to a study by Carolyn P et al., the histopathological evaluation of nine eyes showed no residual tumor in one eye, calcified and necrotic tumor cells in two eyes, and non-calcified, non-necrotic tumor in six eyes.^4^ Demirci et al. studied histopathological features in 10 eyes following chemo reduction. The study revealed regression of the main tumor in all eyes with complete regression in eight eyes and partial regression with viable retinoblastoma in two eyes.^5^

In our study, rosette formation was seen in 33.3% of cases. In a study by Stannard et al., the rosettes were commonly seen in patients with early stages of the disease and thus indicated a good prognosis.^10^ Pseudorosettes characterized by tumor cells surrounding a central blood vessel were also noted in six cases. In our study, extensive necrosis was noted in 52.3% of eyes and extensive calcification was seen in 33.3% eyes. Extensively necrotic retinoblastoma is associated with high-risk histopathological findings like choroidal invasion and optic nerve invasion.^11^ Thus, a thorough evaluation of the eye for the presence of high-risk features is needed in the presence of extensive necrosis. This is significant for eyes without pre-operative chemotherapy. However, its significance in post-chemotherapy-treated eyes has not been reported. In our study, no significant association of necrosis with high risk histopathological features was noted. Other findings seen on histopathological examination included gliosis, foamy histiocytes, hemosiderin-laden macrophages, fibrosis, foreign body type giant cells, and hemorrhage. In one eye, invasion of choroid, optic nerve, sclera and ciliary body could not be determined due to non-visualization of these structures as a result of extensive necrosis. Histologic grade could not be assessed in four eyes due to significant reduction in tumor.

Optic nerve invasion is seen in 25-45% of eyes that undergo primary enucleation.^9^ In our study, optic nerve invasion was seen in 11 eyes (52.3%). According to Shield et al., invasion of optic nerve posterior to lamina cribrosa is associated with high metastatic risk especially if there is simultaneous choroidal invasion.^12^ Choroidal invasion is seen in 12 – 42% of eyes but massive invasion is seen in less than 10 % of eyes.^13^ In our study, massive choroidal invasion was seen in four eyes (19%).

We also correlated the presence of co-morbid conditions like glaucoma, buphthalmos, etc with high-risk histopathological features as shown in Table-II. Patients with glaucoma were more likely to have ciliary body invasion, which was statistically significant. A study by Balaguer et al reported that there is an association between comorbid conditions and invasive disease. The study reported that patients with retinal detachment and vitreous hemorrhage had more chances of optic nerve invasion.^9^

The overall survival rate in our study was 76.2 %. The survival rate of retinoblastoma in developed countries is between 95-98% which reduces to 50% worldwide. An Indian study conducted by Gupta et al. reported survival rate of 63%.^14^

Further studies on presence of high risk pathological features in retinoblastoma and their association with clinical predictors, chemotherapy induced changes and ocular co-morbidities are needed from developing countries which would impact on management strategies and improve survival outcomes.

### Authors’ Contribution:

**NY & NZ:** Equal contribution towards the concept of the study and critical revision for important intellectual content;

**SM:** Drafted the work, revised it critically for important intellectual content;

**KA, SJ:** Revised it critically for important intellectual content;

**BK:** Substantially contributed towards data acquisition, analysis, and interpretation; drafted the work;

All authors approved the final version and also agreed to be accountable for all aspects of the work in ensuring that questions related to the accuracy or integrity of any part of the work are appropriately investigated and resolved.
